# The Progress of Research into Flexible Sensors in the Field of Smart Wearables

**DOI:** 10.3390/s22145089

**Published:** 2022-07-06

**Authors:** Yunlei Yin, Cheng Guo, Hong Li, Hongying Yang, Fan Xiong, Dongyi Chen

**Affiliations:** 1 College of Textile, Zhongyuan University of Technology, Zhengzhou 450007, China; guocheng@zut.edu.cn (C.G.); 5340@zut.edu.cn (H.L.); 5721@zut.edu.cn (H.Y.); 6825@zut.edu.cn (F.X.); dychen@uestc.edu.cn (D.C.); 2 Henan Province Collaborative Innovation Center of Textile and Garment Industry, Zhengzhou 450007, China; 3 College of Automation Engineering, University of Electronic Science and Technology, Chengdu 611731, China

**Keywords:** flexible sensor, smart wearable, electronic skin, health detection, sensing mechanism

## Abstract

In modern society, technology associated with smart sensors made from flexible materials is rapidly evolving. As a core component in the field of wearable smart devices (or ‘smart wearables’), flexible sensors have the advantages of excellent flexibility, ductility, free folding properties, and more. When choosing materials for the development of sensors, reduced weight, elasticity, and wearer’s convenience are considered as advantages, and are suitable for electronic skin, monitoring of health-related issues, biomedicine, human–computer interactions, and other fields of biotechnology. The idea behind wearable sensory devices is to enable their easy integration into everyday life. This review discusses the concepts of sensory mechanism, detected object, and contact form of flexible sensors, and expounds the preparation materials and their applicability. This is with the purpose of providing a reference for the further development of flexible sensors suitable for wearable devices.

## 1. Introduction

With the development of technology, intelligent wearable devices that can be integrated into the human body and perform multiple functions have come from science fiction to people’s real life. With the increasing demand in the field of smart wearables, ordinary sensors have gradually failed to meet the requirements. Flexible sensors with the characteristics of flexibility, foldability, and wearability have become a vital breakthrough [1]. Using flexible sensors, researchers can convert the external force into electrical signals, perform signal processing, and use flexible sensors in wearable products to monitor human body indicators in real-time and accurately [2,3,4,5].

Flexible sensors and traditional sensors are produced with entirely different materials and processes. Flexible sensors must be applied to flexible fabrics, plastic films, etc. They cannot use high-temperature processes, have high requirements for electrical conductivity, and their performance must remain stable under folding, bending, and water vapor infiltration. The properties of flexible sensors vary with the preparation method, and the working mechanism is also different from that of ordinary sensors, including changing the conductive network between overlapping nanomaterials, the tunneling effect, crack propagation, and more [6,7,8]. Due to the various preparation methods of conductive materials, they have greater compatibility with different substrate materials. For example, a wide range of processes, such as dip coating, in situ assembly, mechanical press, transfer printing technology, and chemical deposition, etc., can be used to combine with the substrate [9,10,11,12]. Cyclic stretching or compression is usually used to test the selection of a suitable polymer matrix to design a special conductive network to stabilize the conductive network, so that the sensitive components have a stable sensing behavior. In addition, by means of their bionic function and structure [13], such as artificial nanostructures derived from highly sensitive structures (insect cracks or leaves, etc.) and stretchable structures (wrinkles, textured structures, etc.) that occur in nature, it provides additional feasible strategies for generating good sensitivity and stretchability.

In addition to their continuous mapping capabilities, flexible sensors are characterized by high sensitivity, flexibility, and simple manufacturing processes [14]. However, flexible sensors currently face significant challenges as they are still in the initial stages of development. Flexible sensors must be integrated into object surfaces with compatibility, durability, and wear resistance. In contrast, existing commercial smart wearable devices are mainly implemented by packaging integrated circuits with solid substrates. However, solid substrates are mechanically incompatible with the soft human body, resulting in unreliable measurement results due to measurement position changes and unreliable skin contact.

This paper develops the discussion of flexible sensors in the field of smart wearable from the research history of flexible sensors. Then, the different classifications of flexible sensors in terms of the sensing mechanism, detection mode, and contact form are elaborated. Next, six common materials for flexible sensors are introduced. Next, the frontier applications of flexible sensors in the field of smart wearables are also discussed. Finally, the current challenges and future opportunities of flexible sensors are summarized to provide a reference for the development of wearable, flexible sensors.

### Research History of Flexible Sensors

**Figure 1:**
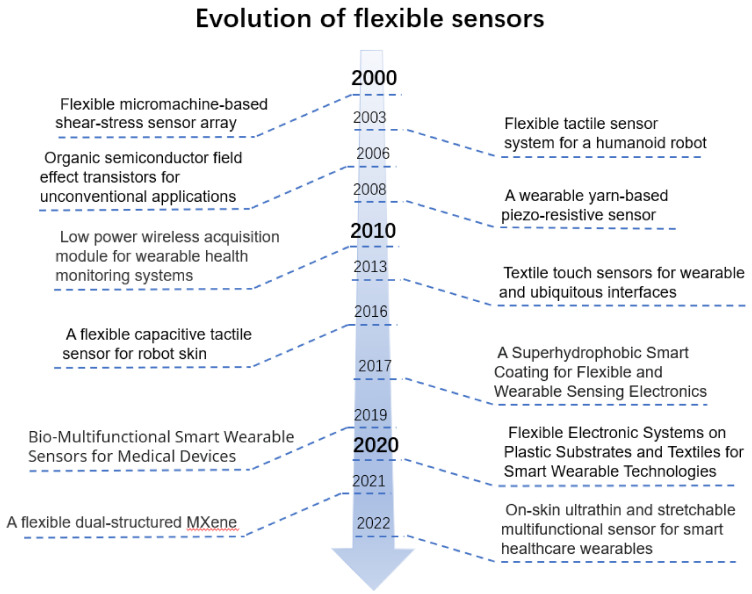
Timeline of the major milestones in the development of flexible sensors in the smart wearable field.

Since the late 1990s, smart wearables have been developed in various ways. More user-friendly and human-centered designs have been developed, such as diverse research on physical factors such as comfort, usability, ergonomics, and wearer design, and smart wearables have been developed in all aspects and are widely used in areas such as electronic skin, health monitoring, sports monitoring, smart medicine, and human–computer interaction. In 2000, Jiang et al. [15] proposed a new microfabrication technique that has been developed and applied to the development of flexible shear–stress sensor arrays. In 2003, Kerpa et al. [16] proposed the development of a flexible tactile sensor system for humanoid robots. In 2006, Manunza et al. [17] fabricated the first fully flexible field effect device for chemical detection based on organic field-effect transistors, which opened the way for the production of flexible chemical and strain sensors. A wearable yarn-based piezoresistive sensor was put forward by Huang et al. in 2008 [18], demonstrating the feasibility of a yarn-based sensor, and the results showed that the yarn-based sensor could accurately track breathing signals. In 2010, Figueiredo et al. [19] developed a low-power wireless acquisition module that does not require the preparation of electrolyte gels, and it provides continuous monitoring of the user’s electrocardiogram (ECG) and activity. The design of textile touch sensing interaction was explored by Roh et al. [20] in 2013 with the new metal composite embroidery yarns (MCEYs) and a simple and easy fabrication technique. In 2016, Ji et al. [21] presented a flexible capacitive tactile sensor for robot skin, and a tactile feedback and signal acquisition system was built for the application of robotic obstacle avoidance. In 2017, Li et al. first fabricated [22] a highly flexible multifunctional smart coating using spray-coating multiwalled carbon nanotubes dispersed in a thermoplastic elastomer solution, followed by treatment with ethanol. In 2019, Wang et al. [23] comprehensively summarized a comprehensive review of the latest progress concerning smart wearable sensors, with a focus on bio-multifunctional (biocompatible, biodegradable, and self-healing) device designs. In 2020, Baeg et al. [24] summarized recent progresses in flexible electronic systems, composed of transistor-based circuitry and active matrices built on plastic or textile substrates. In 2021, Guo et al. [25] developed the design and production of dual microstructures of surface micro-bumps and internal hollow pores in the conductive material, MXene, to obtain a multifunctional high-performance pressure sensor, showing an ultra-sensitive ability to extract gesture behavioral information and physiological information from the sensor signals, and its implications for human health. In 2022, Zhang et al. [26] developed an on-skin ultrathin and stretchable multifunctional sensor for smart medical wearables that paves a promising path for future wearables for smart skin and healthcare applications. The above discussion briefly outlines the timeline of major milestones in the development of flexible sensors in the smart wearable field (Figure 1).

## 2. Sensory Mechanisms of Flexible Sensors

**Figure 2:**
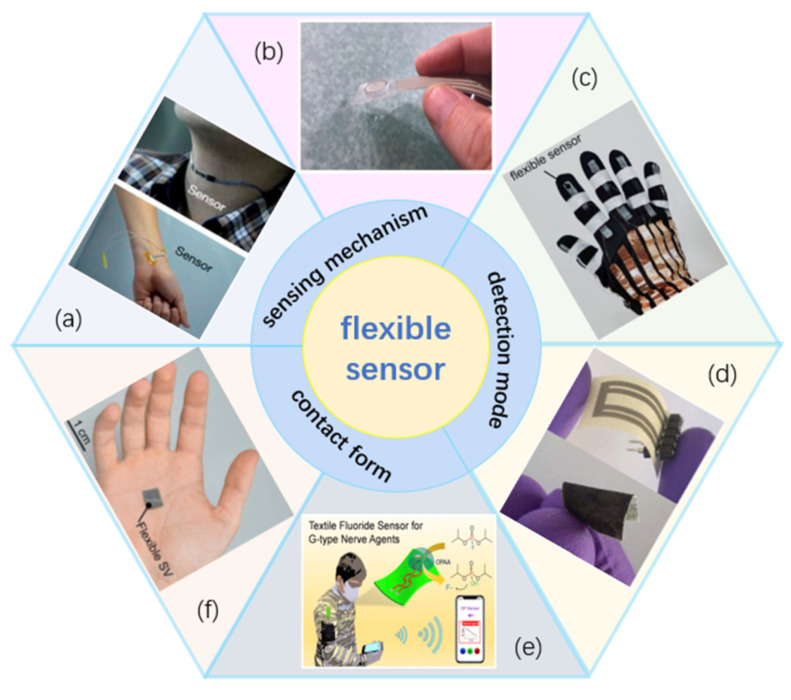
Flexible sensors based on different sensing mechanisms, detected objects, and contact forms: (**a**) a resistive strain sensor attached to the skin [31]; (**b**) a wearable triboelectric sensor for a dynamic prosthetic fit [32]; (**c**) a highly stretchable, tough, and sensitive strain sensor [33]; (**d**) organic chemical sensors for the detection of chemically corrosive vapors [34]; (**e**) contact sensors for bio-detection of flexographically printed fabrics made of fluorine-containing G-type nerve agents [35]; and (**f**) a non-contact sensor for a physiological monitoring system based on the wearer’s palm [36].

Flexible sensors are the focus of intelligent wearable research. They are flexible sensors with different sensing mechanisms, different detected objects, and different contact forms (Figure 2). Flexible sensors can convert external activity (such as mechanical deformation) into directly measurable electrical signals. Depending on their sensory mechanism, they can be divided into resistive [27], piezoelectric [28], capacitive [29], and other types of friction [30]. When the external pressure changes, the appropriate resistance value, capacitance value, or voltage value of the flexible sensor changes accordingly. This means sensors can be alternately categorized as physical or chemical sensors as appropriate to the detected object, and further divided into contact and non-contact sensors according to whether physical connection is required. Of these, contact sensors are the traditional type, in which they cause their own deformation through some physical touch and applied mechanical force, which triggers changes to the electrical signal. This is a step in the mode of operation that non-contact sensors can realize with long-distance reading to collect the same information of the measured object, thus achieving the desired function without physical contact.

### 2.1. Resistive Flexible Sensor

**Figure 3:**
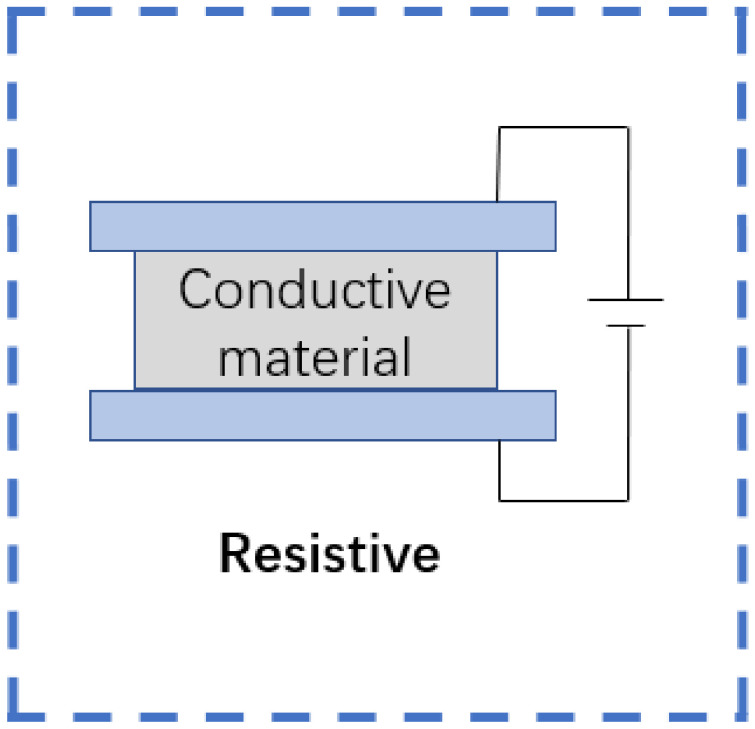
Diagram of the signal conversion mechanism of a resistive flexible sensor.

A flexible resistive sensor is a device that converts external pressure values into resistance or current values. In accordance with the different sensory mechanisms of the device, it can be defined as a piezoresistive or strain type. For flexible piezoresistive sensors, when subjected to external pressure, the conductivity of the conductor material is changed with some small changes in itself [23], i.e., the piezoresistive effect. By the same token, when flexible strain sensors are stretched or compressed by external pressure (that is, when mechanical deformation occurs), the cross-sectional area of the conductor changes, resulting in a corresponding change in the conductive area and thus the recorded resistance [37]. Figure 3 is a schematic diagram of the signal conversion mechanism of a resistive flexible sensor.

A piezoresistive flexible sensor is a sensor based on the piezoresistive effect [38]. When subjected to external pressure, the conductivity of the conductor material changes with minor changes. Flexible piezoresistive sensors have the advantages of a high sensitivity, good linearity, and measurable static force, which has stimulated much research in recent years. Moreover, they can provide a convenient, timely, and portable solution for the detection of motion when worn; however, the development of cost-effective pressure sensor materials with high compressibility and sensitivity has proven to be challenging. Chowdhury et al. [39] developed a flexible and stretchable CNF/PDMS nanocomposite with piezoresistive sensing capabilities via the use of ultrasonic fabrication technology. Excellent electrical conductivity and durable and reliable piezoresistive sensing responses could be obtained by dispersing CNFs into PDMS. The versatility and ease of fabrication of the developed nanocomposites can make up for the lack of high sensitivity and applicability, flaws common to current flexible sensors. This has an undoubted impact on the practical applications of sensors in smart wearables. Yu et al. [40] constructed a highly sensitive, flexible, and wearable flexible piezoresistive sensor based on the structures of pine and needles in nature, in which the sensing layer was composed of graphene-attached tissue fibers and zinc oxide (ZnO). The original 3D structure of the optical fiber was improved by growing ZnO crystals in situ and coupling its piezoelectric capability, and, at the same time, the zinc oxide could be used as a component in the photoelectric sensor. With an ultra-wide testable pressure range (0–100 KPa), the sensor is capable of monitoring subtle pressure changes caused by respiration or the wearer’s pulse (i.e., at the wrist) and large pressure changes that occur during activities such as walking, wrist movements (e.g., from sports), and running. Dynamic or static pressure sensing is achieved thanks to the properties of ZnO and graphene fibers, which also endow wearable sensors with good optical responses. Yang et al. [13], inspired by the surface microstructure of rose petals, developed a layered microstructure as the core of a biomimetic flexible piezoresistive sensor, consisting of a PANI/PVDF nanofiber membrane. This sensor could fit well on the skin, demonstrating it capability for monitoring human physiological signals and motion status, such as wrist pulse, throat activity, spinal posture, and gait recognition. This study provides a promising research scheme for the rapid development of next-generation wearable bioelectronic devices.

As shown by these examples, flexible piezoresistive sensors have attracted great attention in the fields of electronic skin, intelligent robots, and human–computer interaction due to their portability, flexibility, and excellent sensory performance. Li et al. [41] fabricated flexible piezoresistive sensors by treating the backbone of PU with CS to obtain their positively charged equivalents before dip-coating the negatively charged MXene material Ti3C2Tx. The sensors of Ti3C2Tx with CS and PU were observed to provide a versatile sensory platform with the potential to detect large and small pressure signals. Ding et al. [42] also used the dip-coating method to prepare PEDOT:PSS sponges for flexible piezoresistive sensors with good compressibility and a stable piezoresistive response. These were intended for use in the detection of various human actions, including speaking, bending fingers or elbows, and walking. This is indicative of the increasingly important role flexible piezoresistive sensors play in smart wearables, with potential roles in other applications, including virtual reality, entertainment technology, human–machine interface, personal healthcare, and sports science.

**Figure 4:**
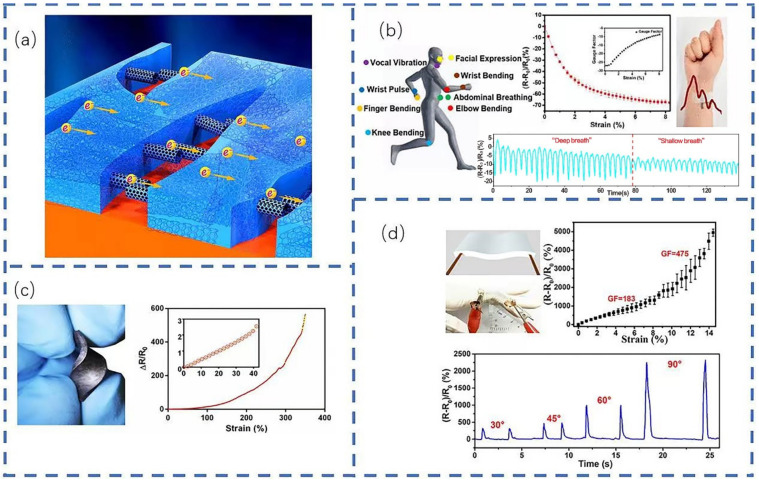
Application of the strain-based flexible sensor in smart wearables: (**a**) a schematic diagram of electron transport at the crack edge in a crack-flexible strain sensor [43]; (**b**) a wearable graphene fabric strain sensor for the detection of various human motions [44]; (**c**) injection of graphene nano-powders into transparent adhesive pads to fabricate flexible strain sensors with an excellent tensile–strain response [45]; and (**d**) the sensory performance in response to the bending of the finger and the degree thereof are both reflected in the relative rate of change in the resistance [46].

Flexible strain sensors can be attached to clothing or human skin for real-time monitoring of human activity. As the core of smart wearable sensing devices, they have generated a great deal of interest among the scientific research community. Among the work carried out in developing them, Zhou et al. [43] developed a sensor with high elasticity and durability by spraying CNT ink onto pre-stretched TPU fiber mats (Figure 4a). This sensor was shown to have a fast response time (70 ms), excellent durability, and good sensory performance when responding to bending. Due to its ultra-high sensitivity, it can easily monitor various subtle movements across the human body. Elsewhere, Yang et al. [44] fabricated a graphene fabric sensor with a negative resistance change by thermal reduction of GO (Figure 4b). Such sensors can be directly knitted onto clothing for monitoring both large and subtle movements. The same team also developed an AgNPs-bridged graphene variant (Figure 4c) for real-time detection of subtle and intense human motion [45]. It demonstrated an excellent range of strain detection with a gage factor (GF) of 475, fast response/recovery speed, and good linearity, showing good durability and long-term stability in stretching/repetitive cycling, compared to the performance of other graphene flexible strain sensors. Larimi et al. [46] fabricated a flexible strain sensor by infusing graphene nanoflakes onto a rubber-like adhesive pad (Figure 4d), resulting in a highly stretchable product capable of withstanding up to 350% strain. Strong and stable electrical responses were maintained even after 10,000 stretching cycles and the sensor showed an ability to measure the human heartbeat and track a wide range of human motion. In other testing, it could also be used in robotic haptic applications to control robotic limbs and extremities.

### 2.2. Piezoelectric Flexible Sensors

**Figure 5:**
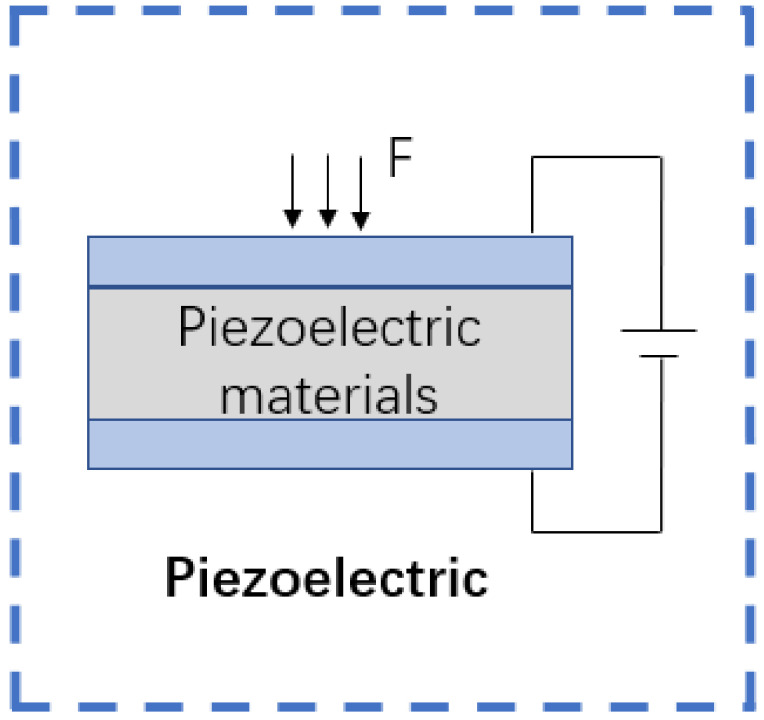
Diagram of the signal conversion mechanism of a piezoelectric flexible sensor.

This type of sensor is designed based on the so-called piezoelectric effect principle of appropriate materials [47]. The piezoelectric effect refers to the deformation (including bending and expansion) of some dielectrics under the action of an external force in a particular direction. In this case, electrical polarization occurs internally, and charges of opposite signs are generated on the two surfaces at the same time. When the external force is removed, the dielectric returns to an uncharged state and when the force’s direction changes, the polarity of the charge also interacts with the changes. The amount of charge generated by the dielectric is proportional to the magnitude of the externally applied force. In this process, the magnitude of the external force can be calculated by the magnitude of the current (Figure 5).

Self-powered physical sensors for wearable applications have been in great demand in recent years, and flexible piezoresistive sensors have taken an important step forward as self-powered devices. Such sensors have the characteristics of self-power, high sensitivity, and good responsiveness [48], and are an important part of the physical movement energy in the human body. Chen et al. [49] developed a flexible triaxial tactile sensor with piezoelectrically enhanced P(VDF-TrFE) micropillars, which has great potential in advanced robotics, wearable electronics, and various human–machine interface implementations. To realize a large-area flexible pressure sensor array applicable for smart wearable products, Zhang et al. [50] developed a PVDF-TrFE/AgNWs piezoelectric capacitor and a-IGZOTFT piezoelectric sensor integrated on a PI flexible substrate in sequence. The doped AgNWs enhanced the piezoelectric properties of PVDF-TrFE while avoiding damage to TFTs, which would have been caused by high-voltage polarization. In addition, combined with the flexible TFT amplification circuit, the corresponding signal was further amplified.

**Figure 6:**
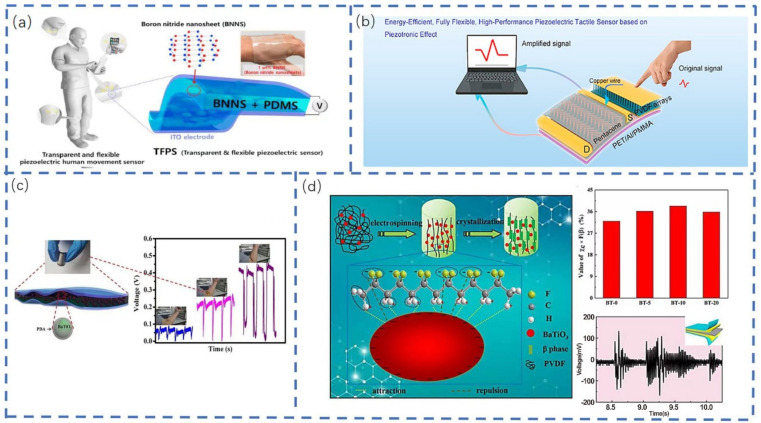
Applications of strain-based flexible sensors in smart wearable sensing: (**a**) a schematic diagram of flexible piezoresistive sensors adapted for human motion, including two layers of ITO electrodes, BNNS/PDMS piezoelectric active layer, and TFPS on the hand device [51]; (**b**) the structure of a high-performance strain-based sensor and a flexible transistor based on the piezoelectric effect, where PET is the flexible substrate and PMMA is the dielectric layer [52]; (**c**) the structure of a high-performance flexible piezoelectric sensor and transistor based on the piezoelectric effect, where PET is the flexible substrate and PMMA is the dielectric layer [53]; and (**d**) the mechanism of electrospinning BaTiO_3_/PVDF composite fibers and the open circuit voltage response to the different sounds of the device vibrating from the speaker [54].

Ultrathin sensing devices utilizing piezoelectric materials have become an important part of developing piezoelectric sensors for continuous bio-signal monitoring systems that can conform to the contours of the skin. Kim et al. [51] developed a transparent and biocompatible BNNS piezoelectric sensor using BNNS and PDMS as a piezoelectric active and flexible element, respectively (Figure 6a). These sensors can generate electrical energy with various signal forms for monitoring body movement. Moreover, Wang et al. [52] proposed an energy-saving and fully flexible piezoelectric sensor (Figure 6b), which was possible through the integration of PVDF nanorod arrays with excellent mechanical force–electrical pulse conversion. These are based on piezoelectric effect-polarized PVDF arrays, which generate sufficient charge to drive the device and realize self-signal amplification, meaning the fabricated sensor has a good pressure sensitivity, detection limit, and response time. Yang et al. [53] introduced PDA as a surface modifier to BTO and blended it with a PVDF matrix in different proportions to form homogeneous PDA/BTO/PVDF composites. From this, a flexible piezoelectric pressure sensor was fabricated using the surface solution casting technique (Figure 6c). The sensor was shown to detect joint bending and human motion exhibited by different movement styles in different signal curves. Additionally, Jiang et al. [54] produced PVDF nanocomposites containing BaTiO_3_ nanoparticles and incorporated them, via electrospinning, into flexible piezoelectric pressure tactile sensors (Figure 6d). These sensors demonstrated a good flexibility and linear response to external mechanical force, and were able to detect different musical sounds.

### 2.3. Flexible Capacitive Sensors

**Figure 7:**
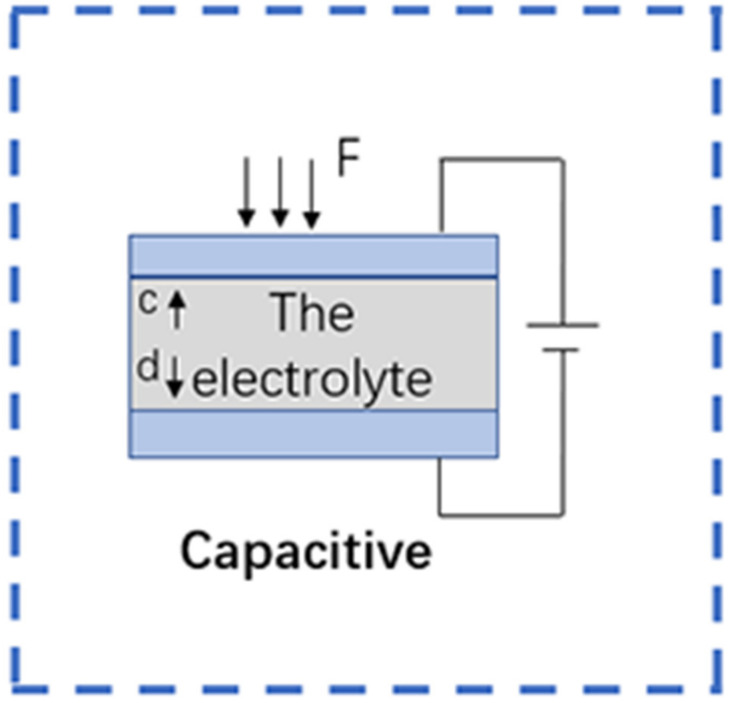
Diagram of the capacitive flexible sensor signal conversion mechanism.

When a flexible capacitive sensor is subjected to external pressure (the magnitude of which can be calculated by measuring the changes in the electrical signals), the capacitance value between the electrode plates also changes [55], causing changes in other electrical signals. Generally, flexible capacitive sensors are dielectric materials with a microstructure added to the middle of the flexible electrode. This microstructure changes significantly under the action of external pressure, which results in changes in the dielectric properties of the dielectric material and thus the capacitance value as well [56]. The sensitivity of flexible capacitive sensors can generally be improved by substituting the material of the dielectric layer, performing related structural modifications, or by compounding the substrate with a substance with a high dielectric constant [57]. Figure 7 shows the signal conversion mechanism of the capacitive flexible sensor.

With respect to wearable electronic devices, capacitive pressure sensors benefit from low energy consumption, good stability, simple structure, and adjustable sensitivity. Unsurprisingly, such developments have attracted much attention, giving rise to further innovations. Xiong et al. [58] made an ultra-high sensitivity flexible capacitive pressure sensor based on convex microarrays and ultrathin dielectric layers. When external pressure was applied to the sensor, the sensitivity of the sensor was recorded to greatly improve due to the synergistic effect of the microarray on the electrode surface and the ultra-thin dielectric layer, resulting in a significant increase in the contact area and a similar reduction in the distance between the two electrodes. This flexible sensor has been successfully used to monitor various human bio-signals and robotic hand movements, paving the way for applications in areas such as smart medical care, automatic speech recognition, and language recognition. Moreover, Li et al. [59] developed a highly sensitive, stretchable, and tough capacitive strain sensor using double-crosslinked zinc alginate-alginate/PAM hydrogels as ionic conductors to repeatedly adhere a PVA/PAA borax organo-gel, which served as the dielectric layer [60]. By combining the mechanical advantages of water/organo-gels, the assembled capacitive strain sensor exhibited high elasticity and excellent sensitivity. These synergistic effects enabled capacitive strain sensors to be used in highly reliable wearable sensory devices to monitor various weak physiological signals and a wide range of human movements, such as finger, wrist, elbow, and knee joint movements, smiling, and talking. Additionally, Keum et al. [61] developed a fabric-based high-sensitivity sensor using silver-coated multifilament fibers woven into a conductive fabric as electrodes and flexible ion-gel membranes with high dielectric constants as sensing elements. The composition of this capacitive system of this sensor allowed it to achieve a multi-point detection capability on large-area fabrics.

### 2.4. Flexible Friction/Triboelectric Sensors

**Figure 8:**
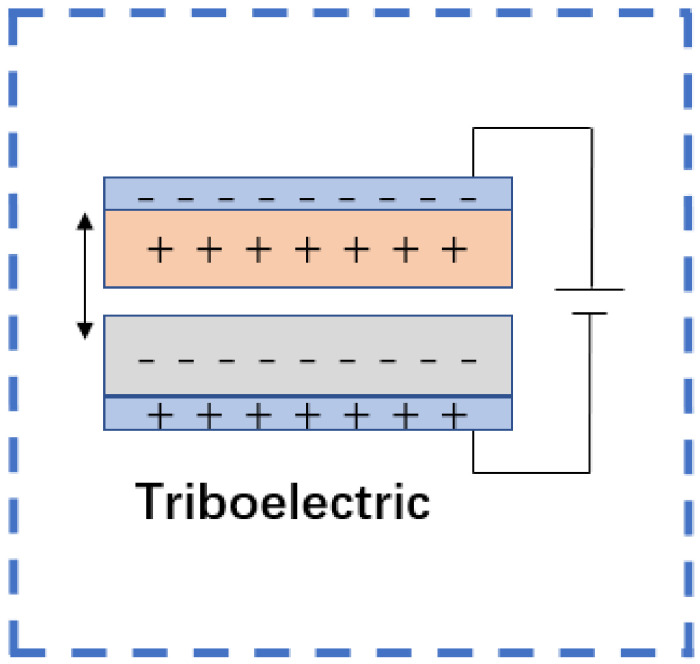
Diagram of the triboelectric flexible sensor signal conversion mechanism.

Flexible triboelectric sensors are typically composed of upper and lower electrodes, each lined with material on the inner face responsible for triboelectric generation, with a thin layer of air between the two. When pressure is applied, two materials with different charged sequences come into contact with each other, and a triboelectric phenomenon occurs, generating opposite charges on both sides of the interface. When the pressure is released, the two surfaces with equal and opposite charges are automatically separated. Due to the phenomenon of electrostatic induction, compensating charges are proportionately generated on the surface of the electrodes, but the air layer between the materials cannot completely neutralize the charges on the two surfaces, resulting in a potential difference [62]. This mechanism enables flexible triboelectric sensors to generate signals when pressure is applied and released (Figure 8).

Mechanical energy, if harnessed efficiently, can not only make a significant contribution to global electrical demands but also serve as an independent and sustainable energy source for mobile electronic devices. However, triboelectricity is difficult to accumulate and utilize, so its value is often overlooked. In recent years, the high efficiency, low cost, and environmental friendliness of triboelectric nanogenerators have attracted attention among researchers. In one instance, Ning et al. [63] constructed a wearable and self-powered triboelectric sensor by introducing a helical structure braided fiber on a stretchable base. Due to this structure, the sensor exhibited high sensitivity, with a single helical fiber-optic strain sensor (10 cm in length) outputting 0.5 V even at 1% tensile strain. For its practical applications, this helical-structured sensor functions by responding to instances of contraction and relaxation of the thoracic cavity and abdomen caused by the heartbeat and breathing. Based on this sensor, a wearable self-powered real-time respiration monitoring system has been developed. Further, Zhang et al. [64] designed a high-performance flexible self-powered sensor with SnS2 restoring GO-based humidity, driven by a triboelectric nanogenerator. Under the effects of external mechanical motion, the nanogenerator generated a large voltage through the triboelectric effect between PTFE and a copper film. Applying this concept to a flexible humidity sensor, an SnS2/GO composite was fabricated on a PET substrate by means of screen printing. By integrating the two components in this manner, a self-powered triboelectric humidity sensor was realized. The sensor featured a high stable output voltage, fast response/recovery time, long-term stability, and ultra-low power consumption, making it suitable for sensing humidity over a wide relative range. In addition, it was capable of monitoring human breathing, coughing, and the approach of objects to the respiratory tract, enabling multifunctional applications in various fields.

The rapid development of robotics and virtual reality technology has put forward higher requirements with respect to advances in the human–machine interface in terms of achieving efficient parallel control. Relevant to this, Zhu et al. [65] proposed a triboelectric bidirectional (TBD) sensor based on the switch and basic grating structures. It had a bidirectional sensory capability for the detection of rotational and linear motions from different joints, including multi-DOF rotation of the shoulder and twists of the wrist. At the same time, the sensor exhibited a good performance irrespective of the speed (good results from 10 to 300 rpm), proving its feasibility in different scenarios. Elsewhere, Chen et al. [66] proposed and studied a self-powered flexible triboelectric sensor patch applied for trajectory and fingertip motion. It was made from environmentally friendly materials, namely starch-based hydrogel, PDMS, and silicone rubber. By designing a grid structure on top of the triboelectric functional layer, only four inductions were needed to detect the trajectory, velocity, and acceleration of the fingertip movements on the 2D-SFTS, and the one-dimensional stretching displacement and velocity could be easily measured. The combination of the two patches achieved the 3D motion control of the robotic manipulator.

Flexible resistive sensors can detect static force and dynamic force with a simple structure but suffer from substantial signal drift, although it is not easily disturbed by external fields. However, they have the advantages of being self-powered and a fast response time. Conversely, analogous capacitive sensors have a simple structure and small signal drift but are easily disturbed by external fields, and their sensitivity is limited by the compressibility of the dielectric layer. Additionally, the flexible triboelectric sensor benefits from a low production cost, simple structure, high output voltage, and self-driving ability. On the measured object, the general resistive and capacitive sensors have a wider range of applications, albeit with exceptions. In some unfavorable environments, the power supply needs to be replaced after some time, which is more labor and material resource intensive, while flexible piezoelectric and triboelectric sensors can detect signals in the field for a greater period of time, saving in costs, thanks to being self-powered. A comparison of sensors with different sensory mechanisms is shown in Table 1.

## 3. Detection Methods of Flexible Sensors

### 3.1. Physical Sensors

Physical sensors are those which use certain conversion element’s physical properties and effects such as dissociation [86], polarization [87], thermoelectricity [88], photoelectricity [89], magnetoelectricity [90], and others. In recent years, these classifications of sensors have been used for signal detection in areas related to smart wearable sensing devices, such as recording body movements, physiological signals, body temperature, and similar such information. External environmental characteristics, for example, ambient temperature and humidity, airflow, and light, are also applicable areas for signal detection.

With the development of advanced materials and fabrication technologies, flexible sensors with multifunctional sensory capabilities are becoming increasingly popular in the field of smart wearables. Furthermore, the combination of elastic substrates with conductive fillers, such as graphene [91], carbon nanotubes [92], metals/semiconductors [93], and conductive polymers [94], has been widely investigated. However, most of these sensors suffer from the disadvantages of a low elasticity, insufficient flexibility, and poor durability due to the rigidity of their materials. This has increased the need for stretchable, flexible, and highly sensitive sensors capable of detecting various mechanically induced deformations. Khalili et al. [95] produced a nanofiber network based on physically cross-linked PLA and TPU blends by electrostatic spinning and depositing a uniform layer of SWCNT on the fibers using a spraying method in order to make the fiber surface conductive. The resulting sensor possessed shape memory properties, wherein the fibers were able to retain their original shape after stretching and thermal recovery. This behavior allows sensors to overcome their plastic deformation and greatly improve their elasticity. Elsewhere, Li et al. [96] developed a multidimensional flexible sensor fabricated from Ag/PDMS composites. The development and production of this type of flexible sensor is simple and fast, and the Ag/PDMS flexible sensor has a variety of applications, including real-time dynamic monitoring of the human body and sound intensity detection. In yet further work, Kumar et al. [97] developed a printable PU nanocomposite ink using PEG as a thermal material, CNT as a heat-resistant conductive filler, and PU as a flexible structural material, using physical mixing and chemical cross-linking methods to develop low-cost, flexible temperature sensors.

### 3.2. Chemical Sensors

Chemical sensors use electrochemical reactions to convert the composition and concentration of inorganic and organic chemical substances into electrical signals. The most commonly used active components are ion-selective electrodes, which serve in this function by measuring the pH of a solution or the concentration of certain ions, such as K^+^, Na^+^, Ca^+^, etc. [98,99]. Currently, chemical sensors are most commonly used in wearable and flexible sensing technologies, which offer advantages such as high sensitivity, miniaturization of the sensors, and direct measurement without markers [100].

Some traditional methods for preparing electrochemical sensors have been applied to the field of smart wearable or flexible platforms. Chemical sensors detect the magnitude of electric currents generated during the oxidation or reduction process of analytes with activity. In this manner, they have been used for the continuous detection of lactic acid, uric acid, glucose, etc., with the help of specific enzymes (e.g., glucose oxidase, lactate oxidase, and urea oxidase) that catalyze chemical reactions of the target metabolite to generate an electric current, allowing the representation of the target analyte’s concentration as the intensity of the generated current. For example, Chen et al. [101] designed and fabricated flexible electrochemical luminescent sensors by immobilizing highly luminescent nanospheres on an AuNT network and coating them with elastic molecularly imprinted polymers. The prepared sensors exhibited continuous and desirable mechanical compliance while generating very stable electrochemically luminescent signals during deformation, helping to detect physiologically relevant chemicals in the body in a highly selective manner. They successfully integrated these sensors into flexible wearable sensing devices, thus providing a promising new method for non-invasive monitoring of metabolites for healthcare and biomedical research. Further, Nakata et al. [99] developed a wearable sweat chemo-sensor for measuring pH in human sweat consisting of an ion-sensitive field-effect transistor integrated with a flexible temperature sensor. Liu et al. [102] prepared a flexible electrochemical sensor for non-invasive urea monitoring with high selectivity in human sweat by electro-polymerizing EDOT monomers on a hierarchical network of CNT and AuNT to imprint template molecules. The sensor successfully analyzed urea levels in external solutions and human sweat with satisfactory sensitivity and high selectivity, providing an effective and promising method for non-invasive monitoring of urea levels in human body fluids.

**Figure 9:**
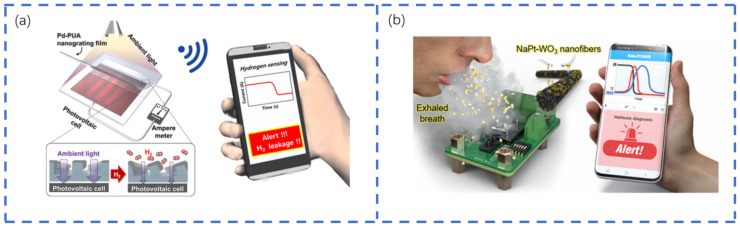
Application of a flexible sensor based on a chemical formula: (**a**) schematic diagram of a self-powered H_2_ gas sensor [103]; (**b**) surface-active tuned metal oxide chemical resistor for direct analysis of human mouth odor [104].

Chemical sensors are sensitive to certain gases, reacting with the tested gas and generating an electrical signal proportional to the gas concentration. The further development and applicability of such functionality have attracted the most interest. Figure 9 shows the application of flexible chemical sensors. Ho Seo et al. [103] developed a self-powered H_2_ gas sensor using a photovoltaic cell and a chemically mechanically deformed Pd-PUA nano-grid structure. The sensor’s mechanism was based on the change in the light transmittance of the nano-grating structure caused by the expansion of Pd upon reaction with H_2_ gas, which was then detected by the change in the photovoltaic cell’s output current. The sensor required no external power source thanks to acquiring its necessary energy from ambient light. It was capable of detecting gaseous hydrogen at concentrations as low as 0.1%; was selective for CO, H_2_S, and NO_2_; and demonstrated good stability against changes in the humidity. Additionally, the sensor exhibited reliability and reproducibility, ensuring its long-term use in practical situations. Meanwhile, Bezdek et al. [104] based SWCNTs on non-covalently functionalized P4VP, a carbon nanotube that doped (Pt-POM) CH4 oxidation catalyst onto the sensor through P4VP coordination, resulting in a significant improvement in all sensor properties. Additionally, Shin et al. [105] performed a simple co-functionalization of WO3 nanofibers with alkali metal (Na) and noble metal (Pt) catalysts by the simple addition of NaCl and Pt nanoparticles followed by electrostatic spinning, which produced a highly sensitive and flexible H_2_S gas sensor. Without any collection or filtration equipment, this sensor offered the possibility of direct, reliable, and fast detection of H_2_S in human breath.

With growing numbers of nanotechnological innovations, the application of material science to flexible electronics has promoted the rapid development of physical and chemical sensors. This has provided a lot of potential for the non-invasive detection of biomarkers in body fluids such as human sweat and saliva, and the diagnosis of physical health-related conditions, a vital part of maintaining and improving people’s standard of living and well-being. A comparison of flexible sensors using different detection methods is shown in Table 2.

## 4. Contact Forms of Flexible Sensors

### 4.1. Contact Flexible Sensors

Contact flexible sensors function primarily via physical contact caused by its own deformation to achieve the detection of stress, strain, and pressure. To obtain and collect this information, deformation can only be monitored in discrete locations and specific routes, meaning the measurement capacity of small-scale strain is limited. They require a certain measuring force to ensure the detection component of the sensor is in contact with the object to be measured and to be fully subject to the changes brought about by the deformation.

In recent years, contact flexible sensors notable for being highly sensitive, stretchable, wearable, and easily integrated into devices have shown great potential and prospects for application in the study of intelligent robots, artificial skin, prosthetic limbs, and portable medical devices. However, they are still limited to recognizing mechanical or mechanical deformations and can currently only sense simple physiological signals such as human pressure, pulse, and vocal cord vibration. Pertinently, Naim et al. [117] developed a novel low-cost, active, dry-contact surface electromyography (sEMG) sensor for a bionic arm. The sensor used stainless-steel electrodes to acquire signals from the surface of the skin, and could be connected to a variety of amplifiers, satisfying the need for an economical and wearable sEMG acquisition system for the development of prostheses. This sEMG sensor and its optimal arrangement was useful for achieving highly accurate individual finger control for the bionic arm and did so relatively cheaply. In a separate development work, Yao et al. [118] proposed a flexible thin-film hand sensor, designed to reliably measure the contact pressure distribution at the viscoelastic handle interface and detect the contact force generated by the limb grasping the tool at that location. Innovatively, Wang et al. [52] constructed a high-performance, energy-efficient, and fully flexible piezoelectric haptic sensor by integrating piezoelectric materials with the electromechanical conversion function and signal of PVDF. Additionally, Peng et al. [119] prepared a flexible p-GaN film/n-ZnO nanowire via laser peeling and direct hydrothermal growth of the nanowires on a film light-emitting diode sensor array, which yielded a mapped distribution of two-dimensional pressure values. It did so by reading the light intensity of all light-emitting diode pixels in parallel. At the same time, the local compressive strain enhanced the intensity of each pixel composed of GaN/ZnO nanowire heterostructures of light-emitting diodes thanks to the piezoelectric photoelectric effect.

### 4.2. Non-Contact Flexible Sensors

Non-contact flexible sensors are defined as those for which the detection of physical quantities is fulfilled by means of non-direct contact. Related technology is widely used in smart wearables, penetrating all aspects of sensor power supplies, and relevant to signals, their generation, modulation, detection, and transmission, etc. Commonly used non-contact methods include optical and thermal detection, and wireless transmission. They currently use the electronic properties of the atomic structure inherent to the surface on certain nanomaterials (such as carbon nanotubes and graphene), or use high-resolution optical sensors, which can not only achieve high spatial resolution and sensitivity but also full-field sensing within a certain range. Thus, they can effectively improve the comprehensiveness and continuity of monitoring and surveillance structures.

These non-contact flexible sensors have received a great deal of attention due to their great potential in human–computer interaction, health monitoring, and healthcare, and their influence has grown alongside the expansion of technological services (i.e., smartphones and internet of things), with increasing demand in light of the coronavirus epidemic. Chen et al. [120] developed a speckle map analysis for a new fiber-optic non-contact displacement sensor with an accuracy and repeatability of 10 nm and a range of 3–4 m. The fiber-optic sensor had a bandwidth of ±5 Hz and could precisely record target positions at 100 Hz. High accuracy and repeatability were achieved using the sensitivity of the fiber optic scattergram and rapid change in the electric field in the near-field region. Taking an alternate course, Guo et al. [121] developed an artificially innervated foam encased piezoresistive non-tactile sensor with self-healing inspired by the mechanosensory based on innervation. The sensor used a new elastic polymer with a metal particle composite sensitive to the direction of any applied contact force, and to the proximity of the human body. In a further innovation, Zhang et al. [122] formed the first flexible sensor based on a flower-like PANI-coated filter paper using a spraying method. Upon preparation, the paper formed a special interwoven flower-like structure, which provided high permeability and bending stability, enabling rapid non-contact detection of nitro-aromatic explosives. Moreover, the results of Tang et al. [123] showed high humidity measuring behavior in both rigid and flexible substrates with a sensor based on SnS nanoflakes. The sensor was able to accurately monitor human breathing patterns and rapidly recognize fingertip movements, showing great promise for applications in physiological evaluation, portable diagnostic systems, and non-contact interface localization.

In recent years, the ability to exchange information between humans and machines has relied heavily on contact-based mechanisms. However, such interactions can lead to serious problems such as unavoidable material degradation through use and potential pathogenic cross-contamination between users. Therefore, a revolution in contactless human–machine interfacing is essential. In this vein, Lu et al. [124] made a flexible, high-sensitivity humidity sensor and array via electrospun PA. The sensor detected the symptoms of asthma in a non-contact manner by monitoring the respiratory rate in real-time. Coupled with a remote alarm system, it could provide a non-contact interface for administering medication to bedridden patients. Consequently, the study provided an effective approach to further development of smart electronics for non-contact scenarios. Tang et al. [125] designed a frictional electric touchless screen sensor from a graphene/ITO/PET triple-layer structure for recognizing various gestures in non-contact operation mode using human body electric charges. The sensor was lightweight, flexible, transparent, could be easily integrated into a smart electronic screen, and was effective at detecting various gestures such as the raising or lowering of a finger, fist clenching, opening of the palm, and palm flipping in different directions and speeds. The results compared positively against conventional capacitive sensors. This innovative frictional electric touchless screen sensor is expected to make a significant breakthrough in intelligent human–computer interaction.

Compared to contact sensing, the non-contact type is more convenient in a variety of situations. Non-contact sensors are equally capable of monitoring the human heart rate, respiration, and other physiological parameters without contacting the human body. Further, this method of monitoring can allow the detection of breathing, pulse, heart rate, and other signals without disturbing the normal activities of the human body. At the same time, because nothing needs to be worn by the test subjects, they may not be aware of the measurement process itself during an extensive data collection period, thereby eliminating the influence of the subject’s psychological responses on the test results; therefore, more objective information can be obtained. Table 3 presents a comparison of the numerous flexible sensors that use different materials and methods of preparation. It analyzes several aspects of their practical performance, including the sensitivity factor, effective linear range, and cycling stability. The results demonstrate that there are still challenges in obtaining optimal flexible sensors (i.e., those with high sensitivity and a good effective linear range at the same time).

## 5. Commonly Used Materials for Flexible Sensors

### 5.1. Flexible Substrates

In order to meet the requirements of flexible electronic devices, materials that are thin, light, transparent, flexible, highly stretchable, insulated, and corrosion resistant have become ideal as applicable substrates. Among the common substrate materials are PET, PMDS, PAN, PU, PVA, PI, and textile materials. Flexible substrates are applied based on how they are deformed (common types are bending, plasticity, and uniaxial, biaxial, and ray-like stretching), and how they store and collect energy. Chen et al. [133] proposed an electrochemically assisted deposition method to deposit GO onto crossed finger electrode patterned PET substrates, and after reducing the resulting films using hydrazine vapor, reduced oxide GO films on bridged PET substrates were obtained. This GO oxide film showed excellent electronic properties and an outstanding gas-sensitive performance. Shang et al. [134] prepared a flexible substrate fluxgate based on MEMS technology and used the prepared flexible substrate fluxgate as a current sensor for online measurement. Jia et al. [135] introduced PAN to TPU and, via electrostatic spinning, fabricated a flexible TPU/PAN film. This was followed up with the preparation of a highly conductive and stretchable Ti3C2 MXene/TPU/PAN film by a simple dip coating process. By introducing PAN to the flexible substrate, this group was able to effectively improve the interaction between Ti3C2 MXene and the substrate without affecting the electrical conductivity of Ti3C2 MXene. Li et al. [136] used in situ chemical oxidation polymerization to synthesize PANI and GO-PANIHs hybrids with a rambutan-like hollow nanosphere structure. Based on the GO-PANIH and flexible PET substrates, the prepared sensors could detect NH_3_ at room temperature without additional electrodes. Moreover, Lin et al. [137] successfully fabricated a flexible and highly sensitive LSG/Cu-NPs glucose sensor using DVD laser-scribed graphene as the conductive substrate. Song et al. [138] synthesized a sponge-like sugar cube PDMS scaffold as a template and developed a porous structure CNT/PDMS sponge with high electrical conductivity and mechanical properties through the drip-drying process of CNT ink dispersion. The CNT–PDMS sponge has high sensitivity as a piezoresistance sensor, which is capable of detecting various stress and has high sensitivity. Moreover, he also developed a highly compressible integrated system comprising a piezoresistance sensor and a compressible supercapacitor based on the wearable CNT/PDMS sponge. Wu et al. [139] first mixed CNF and sugar cube particles as a template and waited until the PDMS solidified after pouring it to remove the sugar cubes to obtain a PDMS/CNF composite. This method transfers the CNF that is originally on the surfaces of the sugar particles and embeds it in the pore walls of the porous PDMS, which ensures good adhesion between the CNFs and the polymer. In 10,000 compression-release cycles of 30% strain, PDMS/CNF showed a better performance of good repeatability. Meanwhile, PDMS/CNF also has stable piezoresistive properties, with good linearity under 70% strain. Using PDMS for swelling in organic solvents, it can be used to monitor the movement of human joints.

### 5.2. Metallic Materials

Relevant metallic materials are generally conductors such as gold, silver, and copper, which are most commonly used for electrodes and wires. For the modern printing process, conductive materials are used for nano-inks, including nanoparticles and nanowires, etc. In addition to good electrical conductivity, metal nanoparticles can also be sintered into thin films or wires. Liquid metals are pertinent materials with low melting points, often at or near room temperature. Due to their high electrical conductivity, low toxicity, and excellent mobility, they are becoming highly desirable candidates as flexible sensors. Kawasetsu et al. [140] developed a flexible and soft inductive triaxial haptic sensor using liquid metals as sensing targets. Liu et al. [141] produced a flexible polymer-based photoelectrochemical sensor by layer-by-layer self-assembly on graphene, which more than tripled its photocurrent. Chen et al. [142] investigated the use of liquid metals with different means of adhesion on various material surfaces to print a circuit with certain patterns on thermal transfer paper. It was then transferred to a flexible substrate, leading to a stretchable flexible pressure sensor. Recently, the discovery of infiltrating metal oxide nanostructures with noble metal nanoparticles has opened the door to the fabrication of biosensors for the detection of volatile organic compounds. Almog et al. [143] used CS’s affinity for heavy metal ions and the functional properties of graphene oxide to generate an environmentally benign organic nanocomposite for flexible electrochemical sensors.

Metal nanowires, especially silver nanowires, are considered the best materials for flexible sensors. Even in highly twisted and multi-cycle test experiments, the excellent aspect ratio and good electrical conductivity of metal nanowires can guarantee a good conduction effect. Yin et al. [144] prepared biodegradable conductive AgNW/CNF hybrid nanoparticles by surface solution blending and vacuum filtration. Due to the amphiphilic property of cellulose, AgNW can be uniformly dispersed, and an effective conductive network can be constructed. In order to study the application of AgNW/CNF nanoparticles in strain sensors, a sandwich strain sensor with a typical microcrack structure was prepared using the pre-strain technique. Due to the different crack densities of structures under different prestrains, interesting and typical prestrain-dependent strain sensing behaviors are observed. Mixed nanoparticles as temperature sensors also exhibit stable and reproducible negative temperature-sensing behavior. This study guides the fabrication of flexible and biodegradable sensors. Shi et al. [145] manufactured a flexible transparent capacitive pressure sensor based on a patterned microstructure AgNWs/PDMS composite dielectric layer material. The dielectric layer material was modified to improve the sensitivity of the device. By mixing AgNW with PDMS at different concentrations, the optimal doping ratio of AgNW was 0.12 wt%. Then, the dielectric layer was microstructured, and a symmetrical PET/ITO film electrode was used. The patterned microstructured sensor had a higher sensitivity (0.831 kPa^−1^), a lower detection limit (<0.5 kPa), and good stability and durability compared to a pure PDMS dielectric layer with a planar structure.

### 5.3. Organic Materials

Large-scale pressure sensor arrays are important for the development of future wearable sensors. Those based on piezoresistive and capacitive signal mechanisms suffer from signal crosstalk, which leads to inaccurate measurements, one of the biggest challenges for wearable flexible sensors. To address this, high-performance organic crystalline materials are considered as strong candidates for the next generation of flexible electronics to be used in displays, image sensors, and artificial skins, amongst others [146]. Not only do they offer flexibility, molecular diversity, low cost, solution processability, and inherent compatibility with flexible substrates, but they also have few grain boundaries and defects, ensuring excellent and uniform electronic properties [147]. Moreover, such materials can be used as a powerful tool to explore the intrinsic electronic and mechanical properties of organic matter, reveal the physical properties of flexible devices, and provide further guidance for the future of the materials themselves and the design of the devices that use them. Lai et al. [148] developed the organic charge-modulated field-effect transistor, a new structure for sensors capable of operating at low voltages, which was shown to be fully applicable across a large swathe of technologies, e.g., from inkjet printing to chemical vapor deposition. Meanwhile, Matsui et al. [149] investigated recent advances and developments in flexible and printed organic thin film transistor devices, including organic materials, fabrication processes, electronic devices, and integrated circuits, highlighting their applications in medical sensors.

### 5.4. Inorganic Semiconductors

Inorganic semiconductor materials have excellent electrical properties but suffer from being brittle and exhibit poor machinability and deformability. Conversely, organic semiconductors are flexible but have generally poorer electrical properties. Researchers have been able to achieve a certain degree of flexibility by preparing inorganic semiconductor films on substrates to reduce the material stiffness, when combined with a suitable structural design. However, this method does not change the intrinsic brittleness of inorganic semiconductors, and there are still major limitations in the processing, fabrication, and applications. Therefore, inorganic semiconductor materials represented by ZnO and ZnS have shown promising applications in the field of wearable flexible electronic sensors due to their excellent piezoelectric properties. Zhang et al. [150] developed an ultrasensitive flexible room temperature gas sensor for lung cancer monitoring and diagnosis based on Na-doped ZnO/reduced GO. Shirley et al. [151] devised a textile pressure sensor using a sandwich model of the semiconductor material ZnO placed between two conductive fabric electrodes. The sensor could be used for a variety of applications in the clinical setting and for tracking human motion. Depending on the target application, the sensor pressure range and sensitivity could be adjusted and optimized while the use of textiles provided a strategy for the effective development of further flexible fabric-based health monitoring sensors. Sholehah et al. [152] successfully fabricated ethylene gas sensors using ZnO-Ag layers on flexible PET-ITO substrates, deposited via electrochemical techniques. Guan et al. [153] built on the flexible ZnO/PAN/nonwoven nanocomposites to produce a gas-phase poly combined with a high-sensitivity NH3 gas sensor. Additionally, Chen et al. [154] developed a watch-type wearable NO_2_ sensor whose core material was ZnS nanoparticles/nitrogen-doped reduced oxide GO. It had a low power consumption (0.52 μW), a low detection limit (69 ppb), and was capable of maintaining its mechanical durability. This sensor provided timely monitoring and early warning of NO_2_ leakage by reading and analyzing signal data via wireless Bluetooth transmission.

### 5.5. Carbon Materials

Carbon materials commonly used for flexible wearable electronic sensors include graphene and carbon nanotubes. The former is light, thin, transparent, has good electrical and thermal conductivity, and has extremely important and promising applications in sensing technology, mobile communication, information technology, and electric vehicles. Mamleyev et al. [155] used optimized laser radiation to pattern graphitic carbon structures onto micron-scale PI sheets and induced rapid local pyrolysis to produce a flexible device. The resulting laser carbon films displayed an excellent electrical conductivity and high surface area with a graded porosity distribution along their cross-section. Qi et al. [156] prepared self-supported flexible enzyme-free sensors by electro-depositing PPy on the surface of flexible carbon paper. This was followed by hydrothermal growth of CoS nanospheres, leading to a final sensor that could be applied to glucose detection in humans. Patil et al. [157] used fine gold nanoparticles doped with porous carbon as electrode materials to fabricate flexible biosensors with high accuracy and reliable operation for the detection of pH and uric acid levels in body fluids. Zhang et al. [158] developed a freestanding flexible film made of graphene/EC nanocomposite, which was integrated with a PET substrate. This gave rise to a highly sensitive flexible gas sensor with an ultra-low strain response. Li et al. [159] prepared flexible fork finger electrodes using silver nanowires, with reduced GO serving as the gas-sensitive material. Li et al. [160] fabricated a novel heterostructure enhanced sensitivity stress/strain sensor consisting of 2D graphene and 1D PZT nanowires as sensitive materials. The constituent carbon nanotubes were characterized by a high crystallinity, good electrical conductivity, large specific surface area, microporous size that could be controlled by the synthetic process, and specific surface utilization up to 100%. Concerning functional nanomaterials, MWCNTs are widely used strain-sensing polymer composites. Due to their unique one-dimensional structure, they are expected to exhibit high anisotropy when aligned along a certain direction, which has a positive effect on improving the sensory performance of pristine polymer composites. Building on this, Laing et al. [161] demonstrated a scalable contact that could be easily integrated into a resistance-based pressure sensor based on CNT conductive networks and photo-resistant insulating layers. Additionally, Hao et al. [162] developed a flexible strain sensor with nanocomposites filled with three-dimensional CNT foam while Huang et al. [163] prepared a flexible lightweight CNT/TPU pressure sensor with a novel aligned porous structure, which demonstrated excellent compressibility and stability.

### 5.6. Two-Dimensional Materials

Two-dimensional nanomaterials have many unique physical properties, high mechanical flexibility, a large specific surface area, and good electrical conductivity, making two-dimensional nanomaterials a good choice as conductive sensitive materials for piezoresistive sensors. Two-dimensional nanomaterials can be widely used in flexible sensors by adjusting their structure, lamellar thickness, and surface modification [164]. These include TMDs and MXene. TMDs refer to compounds formed by transition metals and sulfur atoms and are widely used in molybdenum disulfide (MoS2), vanadium disulfide (VS2), tungsten disulfide (WS2), tungsten diselenide (WSe2), etc. Park et al. [165] studied a large-area tactile sensor based on an active matrix and utilization of the semiconductor and mechanical properties of MoS2. The sensor can measure pressure from 1 to 120 KPa, which is far better than the sensing range of human skin. In addition, the sensor has the advantage of multi-point susceptible detection, which can accurately identify the object’s shape grasped by the human hand by simultaneously monitoring the external pressure in different positions. Yang et al. [166] prepared a high-performance pressure sensor based on 1TMoS2 in PDMS foam as the conductive active layer and layered micromesh veins as spacers. Due to the excellent electrical conductivity and novel structural design, 1Tmos2-PDMs perform better than previously reported devices. It has a high sensitivity of 1036.04 KPa in a wide linear range of 1 to 23 KPa, a fast response time (<50 ms), an ultra-low detectable pressure limit of 6.2 Pa, and excellent repeatable loading and unloading stability (10,000 cycles).

MXene has excellent electrical conductivity, chemical stability, mechanical flexibility, and hydrophilicity. The electrical and optical properties of MXene can be further optimized by appropriate surface modification and functionalization to expand the application range of MXene in flexible wearable sensors. Yue et al. [167] developed a 3D hybrid porous MXene sponge piezoresistive sensor using a dip-coating process and PVA nanowires as spacers. The large contact area between the MXene and sponge and the strong van der Waals force mean that MXene could be fixed on the sponge well. Due to the sponge’s rich porous structure, the sensor showed good compressibility and could be fully restored to its original state after more than 95% of the volume compression. The MXene sponge sensor had high sensitivity over a wide pressure range, low detection limits, fast response times, and excellent durability over 10,000 cycles. Chen et al. [168] prepared lightweight MXene-based aerogel by connecting MXene(Ti3C2) nanosheets into continuous wavy sheets using bacterial cellulose fiber as a nano-binder. The aerogel’s ultra-high structural stability allowed it to withstand 99% extremely high strain over 100 cycles and long-term compression at 50% strain over at least 100,000 cycles. In addition, it has high sensitivity, high linearity, and a low detection limit for minor strains and pressures. Yang et al. [169] spun PU dispersion into stretchable PU felt. Then, the MXene solution was dropped on PU to form the Mxene-PU interlocking conductive network, and then the double-sided carbon adhesive was used to lead the wires at both ends of the network to prepare the MXene/PU strain sensor. When subjected to tensile stress, adjacent MXene sheets covering PU slide between each other, and the internal conductive path is disconnected or connected, resulting in a change in the output resistance. The strain detection range of the sensor is 1~100%, which can be used to detect a pulse, heart rate, finger bending, arm bending, and other human activity signals.

## 6. Applications of Flexible Sensors

Electronic devices of the future will become even more miniaturized, lightweight, and versatile. Without interfering with human movement, they can be centralized in our clothes or on our skin to detect changes in health-related conditions and human movement. These applications are highly coordinated, thus stimulating research interest in making them widely applicable to electronic skin, health monitoring, motion monitoring, smart healthcare, and human–computer interaction.

### 6.1. Electronic Skin

Electronic skin (or e-skin) is a new type of flexible wearable sensor that is transparent, soft, and thin like human skin. By attaching e-skins to the fingers and arms of robots, robots gain the ability to sense external physical contact. Inspired by human skin, many have worked to reproduce haptic functions, but e-skin is needed not only to gain tactile perception but, more importantly, to also differentiate multiple stimuli simultaneously to further gain multidimensional perception. E-skin is the core of the future wearable electronic device network, which has gained wide attention in research into human–machine interface, robot smart skin, medical monitoring, and bio-integrated devices due to its multi-functionality, ultra-thinness, low or even zero energy consumption, flexibility, and biocompatibility. Compared with traditional rigid, brittle, silicon-based sensors, e-skins, which do not suffer such flaws, can effectively capture high-quality signals on curved surfaces due to their elasticity. E-skins are expected to play an important role in the era of intelligence ahead.

**Figure 10:**
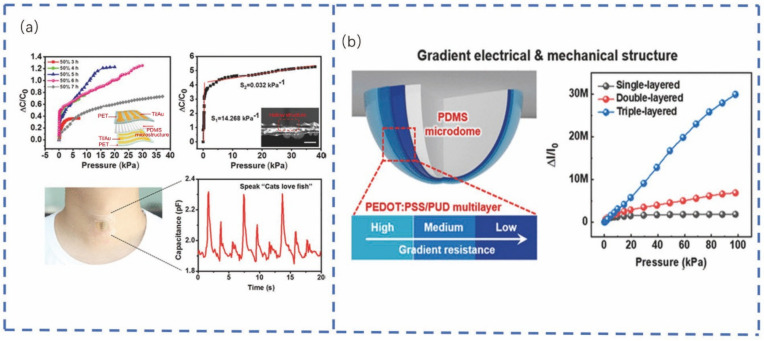
Application of flexible sensors on e-skin: (**a**) a schematic diagram of a flexible pressure sensor based on a wrinkled microstructure with relative capacitance change as a function of the pressure, mounted on a human throat for vocal recognition tests [171]; (**b**) a multilayer PEDOT:PSS/PUD located on the top of a micro-dome pattern PDMS layer with different layers of pressure-related relative current changes in a multilayered electronic skin [172].

Dong et al. [170] designed a stretchable and washable skin-inspired friction nanogenerator for biomechanical energy accumulation and multifunctional pressure sensing. Elsewhere, Zeng et al. [171] developed a tunable pressure sensor with ultra-high sensitivity based on folded microstructures (Figure 10a). By changing the aspect ratio and amplitude of the microstructure in a controlled manner, the sensitivity and operating range could be adjusted, respectively. The sensor achieved a maximum sensitivity of 1.713 KPa^−1^. With a highly compressible microstructure, it also exhibited a relatively low detectable pressure limit (1.5 Pa), fast response time (<50 ms), wide pressure range, and excellent cycling stability. Lee et al. [172] developed a multilayer flexible pressure sensor by simultaneously controlling the tactile stress delivered to the active sensing region and the corresponding current through the gradient structure (Figure 10b). The sensor had an ultra-high level of sensitivity (3.8 × 10^5^ Kpa^−1^) and a very wide pressure range, spanning up to 100 KPa. Additionally, the multilayer pressure sensor was observed to have a fast response time (0.016 ms) and a low minimum detectable pressure (0.025 Pa). This sensor successfully demonstrated diverse applicability, from detecting sounds to weak air pressures, and from mapping pressure in general to monitoring individual health.

### 6.2. Health Monitoring

Lightweight and wearable flexible electronic devices are essential for personal healthcare systems and are not constrained by time and space. In order to monitor human biosignals in a non-invasive manner, highly sensitive, reliable, and sustainable healthcare monitoring devices are required. Highly sensitive sensors can detect small skin strains due to blood flow, pulse, and respiration, and digitize this information for an analysis the subject’s health, such as blood pressure, respiratory rate, and heart rate.

**Figure 11:**
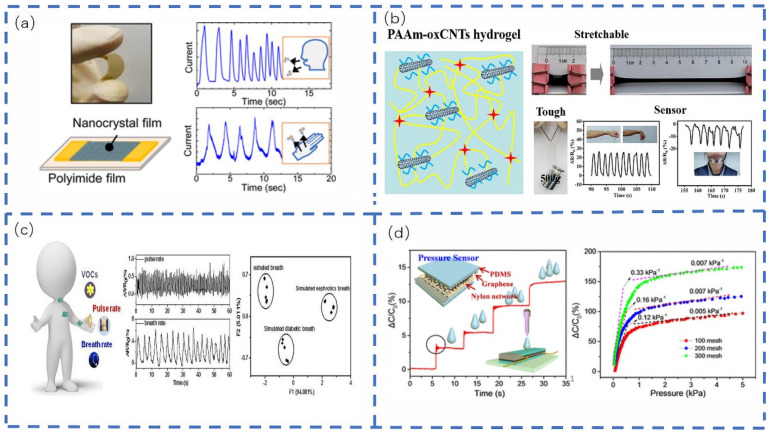
Flexible sensors for health monitoring: (**a**) a schematic of bent forked finger electrodes and a humidity sensor on PI film [173]; (**b**) a schematic of PAAm-oxCNTs hydrogel and images under stretching as an application for monitoring subtle human motion [174]; (**c**) a graphene film flexible sensor with different functions for simultaneous monitoring of physiological signals and biomarkers [175]; and (**d**) a schematic of a low-cost nylon mesh pressure sensor with micro-sized square holes and pressure sensing sensitivity curves for three pressure sensors, each with different mesh sizes of nylon [73].

Kano et al. [173] developed a fast-responding nanocrystal-based humidity sensor, which varied the current reading by 5 orders of magnitude over an 8–83% relative humidity range (Figure 11a). The sensor exhibited a fast response to humidity changes with a response/recovery time of 40 ms at 3 Hz. The research team demonstrated its use in monitoring human breathing and moisture evaporation from the skin in real-time. Furthermore, Shi et al. [69] proposed a simple and low-cost method for fabricating flexible piezoresistive pressure sensors with a large area layered structure. Sun et al. [174] assembled a conductive nanocomposite hydrogel composed of oxidized MWCNTs and PAM (Figure 11b). Xu et al. [175] studied and produced a multifunctional flexible sensor based on two different graphene films, which can detect physiological signals and biomarkers simultaneously without mutual interference. The sensor detects different human physiological signals, such as the pulse and respiratory rate. It has an excellent detection ability by directly attaching to different human body parts (Figure 11c). He et al. [73] developed a cost-effective nylon mesh for a simple capacitive sensor (Figure 11d), which incorporated a nylon mesh with regularly distributed microscale square holes. This sensor exhibited high sensitivity up to 0.33 KPa^−1^ in the low-pressure range and continued to do so after more than 1050 repetitive cycles at 400 and 1000 Pa. This confirmed its long operational durability and an ultra-low detection limit below 3.3 Pa, which can be applied to real-time monitoring of the human pulse.

### 6.3. Motion Monitoring

The data obtained by those flexible sensors applied to human motion monitoring is relatively simple, predominantly based on basic flexion of the limbs or extension movements. Consequently, the data analysis in sports science or fitness has not been fully brought up to date with real scientific requirements, and the data mining, comprehensive analysis, and means of utilization need to be continuously improved. In addition, motion monitoring is a time-consuming process in terms of output and feedback, especially for the module of data collection and transmission. Their working mode is often constrained by the battery’s power reserve, with an insufficient energy supply adversely affecting the accuracy and stability of the collected information.

**Figure 12:**
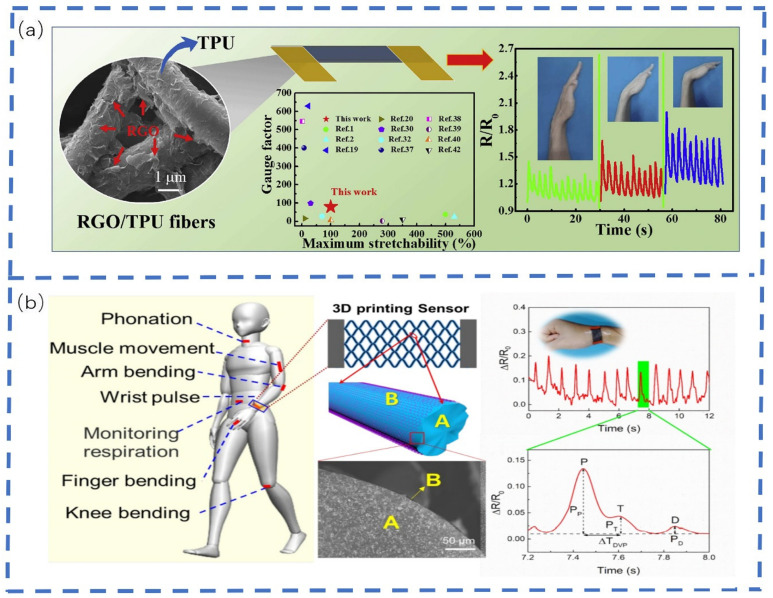
Application of flexible sensors for motion monitoring: (**a**) electron microscopy images of the surface and cross-section of the strain sensor and response curves on the wrist at different curvatures [176]; (**b**) self-compensating second-order structure high-sensitivity and large range strain sensor for human motion detection and its sensing position and corresponding overview [180].

Wang et al. [176] devised a flexible resistive strain sensor with a special three-dimensional conductive network using a flexible TPU fiber mat decorated with reduced GO (Figure 12a). This strain sensor demonstrated excellent electrical properties, hyper-elasticity, high sensitivity, good durability, and fast responsiveness. The full range of human motion detection (including large and fine human motions such as walking, jumping, finger bending, coughing, vocalization, and fine muscle movements of the cheeks) indicated this sensor had a wide range of applications. Using the solution method and ultrasonic dispersion technology, Yang et al. [177] prepared a room-temperature RTV silicone rubber based on CF and CB with excellent elasticity and high electrical conductivity of rubber composites. The sensitivity properties and strain sensing mechanism of CF/CB-RTV silicone rubber sensors were investigated, and it was concluded that the prepared composites have great potential as smart monitoring strain sensors and flexible electrodes. This study thus provided an important alternative solution for the fabrication of low-cost and high-performance strain sensors and electrodes based on conductive elastomer composites. Ergen et al. [178] developed an aerogel-based strain and sweat sensor able to efficiently extract real-time information by combining involuntary human motion and chemical signals. These sensors possessed good mechanical integrity, allowed high-density electrical energy generation during fine body movements, and could be applied to continuous human motion monitoring by measuring the pH, ion concentration, and volume of secreted sweat. Pu et al. [179] fabricated sensors for targeted detection by oxidation of a Ti3C2Tx MXene surface to form nanocrystalline TiO_2_, which were surmised to function by forming a sensor with strongly interacting sensing layers. The strain off-domain was efficiently converted into strain localization by introducing a loose isolated network structure in the sensing layer and two distinct sensing layers with microcrack and through-crack patterns were easily fabricated. The sensors exhibited an excellent performance, including flexibility, fuzzy drift, frequency stability, fast response and relaxation time, and long-term durability. It was concluded that it is highly feasible to achieve a full range of human motion monitoring demonstrations by selectively using sensors for targeted deformation detection. Ma et al. [180] manufactured a strain sensor with high sensitivity and an extensive working strain range based on 3D printing technology and plasma processing (Figure 12b). The concept of a self-compensating second-order structure is proposed for the first time to balance the sensitivity within the strain range (0–350%). At strains below 20%, the sliding and breaking of the graphene sheet coated on the grid surface dramatically improved the sensor’s sensitivity, up to 20 times as much as that of the non-second-order structure sensor. This unique structural design enables sensors to detect the full range of human motion, from pulse and acoustic vibration to breathing and arm bending, providing a new approach for the development of a new generation of full-range strain.

### 6.4. Smart Healthcare

The creation of new intelligent medical wearable products that feature flexible sensors is an important step for promoting the continuous development of advanced healthcare. Through big data, cloud computing, and other technological applications, sensors collect a large amount of data concerning users’ health, behavior, and habits in real-time, providing important support for medical analysis. Matching this, internet-based medical and health data systems are rapidly evolving, allowing doctors to develop timely and effective healthcare plans based on the analysis of routine patient examinations and clinical studies. To obtain this data accurately, continuous and repeated measurements must be taken with wearable devices, which can be used for monitoring patients when they exercise or sleep. Combining medical systems with wearable sensors [181] and applying big data to the construction of medical platforms will allow for the effective analysis of large quantities of recorded information.

Xu et al. [182] designed an adjustable external fixator for the lower limbs, which was able to realize timed intelligent temperature control and meet local fixed-point cooling. The adjustable structure and application of an intelligent air cushion was hypothesized to meet the adequate fixation of the lower limbs of individuals with varying statures and solve the problems of emergency transportation and subsequent treatment of related injuries, such as those commonly suffered by winter sports athletes. Liu et al. [183] introduced a wireless sensor network into a healthcare system and proposed a new form of imaging system. Feng et al. [184] developed a motion monitoring technique for flexible integrated sensor systems that is able to independently measure bending strain and pressure coupled with a low-cost and simple manufacturing process. Yu et al. [185] prepared ultra-flexible and lead-free piezoelectric nanogenerators as highly sensitive self-powered sensors for monitoring human motion, which displayed promise for applications in smart healthcare.

### 6.5. Human–Computer Interaction

Human–computer interaction refers to the exchange of information, via a number of methods, between a user and a digital system, whether it be a machine or software. With the development of computer technology, the interactive devices people use are no longer limited to just keyboards or mice but can also be smart wearable sensing devices.

Wan et al. [186] posited a multimodal artificial sensory memory system consisting of sensors to generate bionic visual, auditory, and tactile inputs, and flexible carbon nanotube synaptic transistors with synaptic-like signal processing and memorization behavior. Zhao et al. [187] developed pressure sensors based on fabricated flexible organic friction transistors with a high sensitivity, fast response time, and excellent stability. Liu et al. [188] proposed a multifunctional dual-network composite hydrogel with good biocompatibility, tensile, and self-healing properties, which showed great potential for human–computer interactions.

In recent years, the field of human–computer interaction has faced great challenges, and high sensitivity, accuracy, reproducibility, mechanical flexibility, and low cost have become requirements for new products that might be in general use. Recent advances in soft materials and system integration technologies offer a unique opportunity for their use in advanced healthcare and human–machine interface design. The hybrid integration of biocompatible materials with miniaturized, wireless wearable systems is an attractive prospect.

## 7. Conclusions

Over the past few years, many researchers have researched and made significant progress in the improvement of flexible sensors. Challenges in terms of accuracy, reliability, high power consumption, rigidity or large volume factors, and difficulties in data interpretation have limited their wider application. Flexible sensors that manage to meet the challenges of high sensitivity, high accuracy, high reproducibility, high mechanical flexibility, and low cost are urgently needed for smart wearable sensing devices to enable their practical application in areas such as e-skin, health monitoring, motion monitoring, smart healthcare, and human–computer interaction. On this basis, the focus should be placed on three aspects of flexible sensors: sensory mechanisms, the subject of detection, and the form of contact, with the hope of providing references for the research and construction of new high-performance structural sensors.

However, despite great advances in the research on flexible sensors, new sensing mechanisms and types of microstructure design should be further explored to develop them with an even better overall performance. At present, particular performance issues (i.e., achieving high sensitivity and a large scale of monitoring at the same time) still need to be solved. In addition, their practical application on the human body or in harsh environments is more limited, so the development of more environmentally friendly sensors is the focus of future research. As more research is conducted, more functions will open for flexible sensors, including self-healing and biocompatibility, etc. The main problem in the future will be how to integrate multiple sensing functions and power sources into a single intricate device. An example of this is a multifunctional system similar to human skin, which requires the incorporation of multiple electronic components and sensors onto very small soft substrates, thus requiring large-area, low-cost integration and manufacturing techniques. At the same time, multifunctional sensors will generate a large amount of electrical signal information. Thus, the matter of collecting and maintaining the accumulated data, and ensuring the functionality and long-term stability of integrated sensors need to be further explored. Therefore, it may be safely concluded that such sensors will become mainstream in the next generation of smart wearable sensing devices.

## Figures and Tables

**Table 1:** Comparative study of flexible sensors with different sensing mechanisms.

Classification	Material	Preparation Method	Linear Range KPa	Sensitivity KPa^−1^	Cyclic Stability (Times)	References No.
Flexible piezoresistive sensor	MXene, Polyacrylate	Coating Techniques	0–16	148.26	13,000	[67]
	TPU, C-MWCNTS	Electrospinning	0–10	2	1000	[68]
	PDMS, Graphene	Spraying	0–25	1.2	1000	[69]
	PDMS, C-MWCNTS	Laser processing	0.2–30	11.06	1000	[70]
	PDMS, IPA	Laser engraving	20–800	6.417	1000	[71]
	SiO_2_, PANI, PLGA	Electrospinning	10–380	0.071	2000	[72]
Flexible capacitive sensor	Graphene, PDMS	Laser processing	0–600	0.33	1000	[73]
	Graphene, PDMS	Chemical vapor deposition	1–10	3.19	500	[74]
	PDMS, CCP, NaCl	Forming and dissolving	0–10	1.1	11,000	[75]
	PS, GNP, MWCNTs	Dip coating	0–5	0.062	2000	[76]
	PDMS, AgNWs, PET	Spin coating	0–2	2.04	1000	[77]
	GNWs, PDMS, ZnO	Spin coating	0–22	22.3	2000	[78]
	AgNWs, PDMS	Screen printing	0–10	2.1	3600	[79]
Flexible triboelectric sensor	PVA, PEI	Template assist	5–50	0.063	2000	[80]
	PU, AC	Curing print	0–10	0.94	1300	[81]
	TMAH, PDMS, P(VDF-TrFE)	Direct deposition	0–900	2.97	40,000	[82]
	CNT, FEP, PDMS	Spin coating	-	0.21	10,000	[83]
	PAN, C/BTO	Carbon electrospinning	0.15–25	1.12	60,000	[84]
	Organic-based ferrofluids, PEG, PEGDA	-	0–44	6.75	20,000	[85]

**Table 2:** Comparative study of flexible sensors with different detection methods.

Classification	Material	Preparation Method	Linearity Range	Sensitivity	Lower Limit of Detection	References
Physical Flexible Sensors	PVA, NaCl	Water Bath Heating	24.8–587 KPa	-	24.8 KPa	[106]
	AAm, AC, SDS, KPS, TMEDA	Fine emulsion polymerization method	54–935 KPa	-	54 KPa	[107]
	[EMIM]Cl, PHEA, PEGDA	Hybrid heating	0–170 KPa	-	0	[108]
	PDMS, Basic Elastomer	Anisotropic etching	0.05–6 KPa	317.19 KPa^−1^	0.05 KPa	[109]
	Silver Nano, TMAH, PDMS	Synergy of microstructures	1–10 KPa	2 KPa^−1^	1 KPa	[110]
Chemical Flexible Sensors	CuO Nanowires, PET	Thermal Oxidation	0–12 mM	-	0.05 mM	[111]
	PET, ITO	Laser Scribing	PH 4–10	−55 mV/pH	PH 4	[112]
	PVIM, MNC, Co_5_POM	-	1 fM–5 mM	210 µAµM^−1^cm^−2^	1 fM	[113]
	PDMS, Basic Elastomer	Anisotropic Etching	2.00–30.00 mM	10.72 µAµM^−1^cm^−2^	10	[114]
	PLLA–PTMC, AuNano film, polybutylenol film	Electrochemical deposition	0.01–100 μM	-	3.97 nM	[115]
	PEDOT, PSS	Wet spinning	PH 3–7	−56 ± 7 mVpH^−1^	PH 3	[116]

**Table 3:** Comparison of flexible sensors using different methods of contact.

Classification	Material	Preparation Method	KPa Linearity Range KPa	KPa^−1^ Sensitivity KPa^−1^	Cycling Stability (Times)	Reference
Contact Flexible sensors	PEN, PVDF-TrFE	Chemical Vapor Deposition	40–200	0.15	200	[126]
	AGNW, PVP, PET	Sandpaper forming	0–4	0.0328	-	[127]
	PDMS, CIP, PEGDA	Spin coating	0–2	0.301	5000	[128]
Non-contact Flexible sensors	PEDOT, PSS, EG, OTS, PDMS	Spray coating	1–5	0.1	2000	[129]
	PPG, TDI, copper powder	Ultrasonication	1.3–38.2	-	500	[130]
	PVP, TEMPO, AgNW	Wet Spinning	0–460	5.49	1200	[131]
	SiO_2_, Si wafer, SigmaAldrich	Photolithography	0.72–11.6	-	5000	[132]

## Data Availability

Not applicable.

## Abbreviations

Chitosan: CS; Polydimethylsiloxane: PDMS; Carbon Nanofibers: CNF; Two-dimension transition metal carbides, nitrides, and carbonitrides: MXene; Thermoplastic Polyurethane: TPU; Carbon Nanotubes: CNT; Graphene Oxide: GO; Isopropyl alcohol: IPA; Silicon Dioxide: SiO_2_; Polyaniline: PANI; Poly(Lactic-co-glycolic Acid): PLGA; Carbon Conductive Paste: CCP; Sodium Chloride: NaCl; Polystyrene: PS; Graphene Nano: GNP; Multi-walled Carbon Nanotubes: MWCNT; Silver Nanowires: AgNWs; Polyethylene Terephthalate: PET; Zinc Oxide: ZnO; Polyvinyl Alcohol: PVA; Polyethyleneimine: PEI; Polyurethane: PU; Activated Carbon: AC; Tetramethylammonium Hydroxide: TMAH; Polyvinylidene fluoride-trifluoroethylene: P(VDF-TrFE); Single-walled Carbon Nanotubes: SWCNTs; Fluorinated ethylene propylene: FEP; Polyacrylonitrile: PAN; Barium Titanate: BTO; Polyethylene Glycol: PEG; Poly(ethylene glycol) diacrylate: PEGDA; Silver Nanoparticles: AgNPs; Thin Film Transistors: TFT; Boron Nitride Nanosheets: BNNS; Polyacrylamide: PAM; Polyacrylic Acid: PAA; Polytetrafluoroethylene: PTFE; Polylactic Acid: PLA; 3,4-Ethylenedioxythiophene: EDOT; Poly(4-Vinylpyridine): P4VP; Platinum-polyoxometalate: (Pt-POM)CH4; Tungsten Trioxide: WO3; Indium Tin Oxide: ITO; Poly(ionic liquid)s: PVIM; Cobalt Polyoxometalate: Co5POM; Poly(3,4-ethylenedioxythiophene): PEDOT; Polystyrene Sulfonate: PSS; Ethylene Glycol: EG; Toluene Diisocyanate: TDI; 2,2,6,6-tetramethylpiperidinooxy: TEMPO; Fluorododecyl Trichlorosilane: SigmaAldrich; Gallium Mononitride: GaN; Polyethylene Naphthalate: PEN; Polyvinylidene Fluoride: PVDF; Polyvinylpyrrolidone: PVP; Carbonyl Iron Particles: CIP; Ethyl Cellulose: EC; Polypyrole: PPy; Acrylamide: AAm; Sodium Dodecyl Sulfate: SDS; Potassium Persulfate: KPS; Tetramethylethylenediamine: TMEDA; 1-Ethyl-3-methylimidazolium Chloride: [EMIM]Cl; Poly(ethylene glycol) Diacrylate: PEGDA; Poly (2-hydroxyethyl methacrylate): PHEA; Lead Zirconate Titanate: PZT; Transition metal chalcogenides: TMDs; Poly(ethylene glycol) Diacrylate: PEGDA; Room-temperature-vulcanizing Silicone: RTV; Carbon Fiber: CF; Carbon Black: CB.
